# Infant temperament and family socio-economic status in relation to the emergence of attention regulation

**DOI:** 10.1038/s41598-018-28831-x

**Published:** 2018-07-25

**Authors:** Ángela Conejero, M. Rosario Rueda

**Affiliations:** 1 Department Psicología Experimental, Universidad de Granada, Granada, Spain; 2 Centro de Investigación Mente, Cerebro y Comportamiento (CIMCYC), Universidad de Granada, Granada, Spain

## Abstract

Attention regulation refers to the ability to control attention according to goals and intentions. Disengagement of attention is one of the first mechanisms of attention regulation that emerges in infancy, involving attention control and flexibility. Disengaging attention from emotional stimuli (such as threat-related cues) is of particular interest given its implication for self-regulation. A second mechanism of attention control is the ability to flexibly switch attention according to changing conditions. In our study, we investigated 9 to 12-month-olds’ disengagement and flexibility of attention, and examined the contribution of both temperament and socioeconomic status (SES) to individual differences in the emergence of these attention regulation skills at the end of the first year of life. Our results show that both difficulty to disengage from fearful faces and poorer attention flexibility were associated with higher levels of temperamental Negative Affectivity (NA). Additionally, attention flexibility moderated the effect of NA on disengagement from fearful faces. Infants with higher NA and poorer attention flexibility showed the greatest difficulty to disengage. Low SES was also associated with poorer attention flexibility, association that was mediated by infants’ NA. These results suggest that attention flexibility together with temperament and environmental factors are key to understand individual differences in attention regulation from threat-related stimuli as early as from infancy. Our findings also stress the importance of interactions between environmental and constitutional factors for understanding individual differences in the emergence of attention regulation.

## Introduction

The voluntary control of attention is a central aspect of self-regulation^1^. In the attention network model proposed by Posner and Petersen^2,3^, the executive control of attention (i.e. executive attention) is one of the three main functions of attention together with orienting and alerting. At early stages of development, the control of attention is primarily observed over orienting. In the first months of life, orienting of attention is quite inflexible and externally controlled by changes in stimulation. In fact, young infants are often unable to disengage attention from foveated stimuli, even after being sufficiently explored^4^. This is because in the first months of life attention is mainly controlled by the superior colliculus^5^, a brain structure involved in attentional engagement. By 3 to 4 months of age, the maturation of the frontal eye fields and superior parietal lobe brings about the flexible control of the colliculus and thus facilitates the endogenous control of attention^6^. This manifests in babies’ capacity to disengage attention from central stimuli. Therefore, disengagement of attention is regarded as one of the first mechanisms of attention regulation that emerges during infancy. During the second half of the first year of life, infants start to employ disengagement strategies (e.g. looking away from distressful stimuli) in order to down-regulate distress^7^. Thus, the emergence of endogenous control of attention in infancy is thought to build the foundations for more complex emotion regulation skills that will develop in the upcoming years^8^.

In general lines, stimuli involving some degree of threat or fear tend to capture attention preferentially^9,10^. The initial capture of attention by threat-related cues can be seen as an adaptive mechanism that benefits the prevention of a potential menace^11^. However, keeping attention on threatening stimulation may become maladaptive if it impairs the shift of attention to other information of interest. In fact, the difficulty to disengage from threatening stimuli is largely assumed as one of the mechanisms underlying some anxiety disorders^12^. Delays disengaging from fearful faces have been reported in children prone to anxiety^13^. The same pattern has been observed in infants from 7 months of age^14–19^. Infants showing higher negative affectivity or insecure attachment appear to have greater difficulty disengaging attention from fearful faces^16,18^. In adults, neuroimaging data reveal that disengaging from fearful faces (but not from neutral or happy faces) involves the activation of structures within the brain system for endogenous (i.e. top down) control of attention^20^. Thus, differences in attention control have been proposed to explain individual differences in disengaging attention from threat-relevant stimuli^21–24^.

A different form of attention control that has been studied in infants is flexibility in switching attention. Attention flexibility allows the adjustment to situational demands by applying inhibitory control over dominant but inappropriate behavioural tendencies, enabling the selection of more adaptive responses^25^. This function is supported by the maturation of frontal brain structures within the executive attention network such as the dorsolateral prefrontal and anterior cingulate cortices. During the second half of the first year of life, babies start to show some capacity to flexibly change the attention focus and adjust responses according to goals and contingencies. For instance, babies from 9 months of age onwards can inhibit the tendency to reach toward objects in the line of sight to successfully retrieve objects through an open side of a transparent box, or search for a hidden object in a new location that conflicts with a previously rewarded one (reaching and A-not B tasks respectively^26^). Moreover, individual differences in the performance of attention flexibility tasks become apparent during infancy^27^. Despite that, no studies to date have investigated how attention flexibility contributes to incipient individual differences in disengagement of attention from threat-related stimuli in infancy.

Both intrapersonal and environmental factors may have an influence in the development of attention flexibility and disengagement from threat-related stimuli. About intrapersonal factors, research on individual differences in attention and self-regulation have established a close relationship between attention regulation and temperament along development^28^. Temperament refers to intrinsic individual differences in emotional, motor, and attentional reactivity in conjunction with the self-regulatory processes that modulate such reactivity, which are observable from birth^29^. Reactivity is classified along a negative/avoidant (i.e. negative affectivity, NA) and a positive/approaching (i.e. surgency, SUR) axis, whereas regulation has been linked to executive attention^29^.

Some studies have explored the interaction between attention control and temperament. Generally, higher temperamental reactivity and poorer self-regulation are associated with deficits in attention control^30^. In particular, NA has been related to difficulties in regulating attention to emotional stimuli. Infants characterized as high in NA are highly irritable, easily frustrated, show intense fear towards novel stimuli, and take more time to calm down once they are distressed^31^. Also, similarly to adults, infants rated as high in NA usually show poorer attention skills^32,33^, as well as greater difficulty to regulate attention from threat-related stimuli^16^. Likewise, children with fearful temperament and low effortful control show greater attention bias toward threat-related cues^34^, and greater engagement to threatening (angry) faces is predicted for children with poorer performance in attention control and higher levels of NA^35^. These studies indicate that individual differences in attention bias toward threat-relevant stimuli are a result of the interaction between attention control and temperament.

Regarding environmental factors, a growing body of literature suggests that the nurturing environment plays an important role in the development of attention control. It has been shown that children raised in low socioeconomic status (SES) environments perform poorly in tasks involving attention control compared to high-SES children^36,37^. Research has mainly studied the impact of SES on attention during childhood and adolescence with only a few studies exploring the influence of home environment in the development of attention in infancy. Particularly, low-SES seems to have a negative impact on infants’ attention flexibility^38,39^. Disparities in SES may also influence infants’ development of attentional disengagement. Existing research so far did not directly address the relationship between SES and disengagement from threat-related stimuli. There is evidence linking low-SES to poorer attention regulation to emotional stimuli. For instance, children who grew-up in low-SES contexts show a diminished activation of brain structures involved in the voluntary control of attention as adults when asked to regulate emotion to negative valence stimuli^40^. In addition, infants raised in deprived home environments appear to show increased NA, suggesting that low-SES might prompt to higher levels of NA^41^. Alternatively, infants with different temperamental profiles might be more or less influenced by the environment as proposed by the differential susceptibility model^42^.

The goal of the current study was to investigate individual differences in regulating attention to threat-related stimuli during infancy by examining the role of attention flexibility. For this purpose, we measured infants’ ability to disengage attention from emotional faces. We expected infants to show greater difficulty to disengage from fearful faces compared to neutral or positive ones as reported by previous studies^14–19^. We also measured attention flexibility with a switching task. Given their common underlying brain basis, we hypothesized that babies’ performance in both the disengagement and switching tasks would be positively related to babies’ performance in the switching task, with infants showing the greater flexibility also showing better capacity to disengage attention from faces. More specifically, we anticipated that flexibility of attention would be more strongly related to the disengagement from fearful faces, as the regulation of attention to threat-related stimuli may require the control of attention to a greater extent^43^. We further explored the influence of temperamental and environmental factors on this association. To test the effect of SES and temperament on individual differences in attention regulation, we obtained parental reports of family SES and infants’ temperament. Despite prior research suggesting that both temperament and environmental factors may contribute to the emergence of individual differences in the ability to regulate attention, few studies have included the two types of variables to test how they relate in early development. We hypothesized that both SES and temperament would be related to the ability of infants to disengage and flexibly switch attention. Thus, we expected that infants with higher NA and from lower SES families would have greater difficulty to disengage attention from fearful faces and poorer attention flexibility. Following previous research with children^35^, we anticipated that attention control would modulate the association between NA and disengagement from fearful faces. Finally, we explored the relationship between SES and NA to predict such individual differences in disengagement and attention flexibility. Despite existing evidence not allowing to formulate clear hypotheses, we also wanted to explore whether our data would be better fitted by a mediation (the effect of SES mediated by NA) or a differential susceptibility model (NA moderated the effect of SES), in order to explain the expected interaction between temperament and environment variables.

## Method

### Participants

The initial sample consisted of 73 infants between 9 to 12 months of age (34 males, 39 females; mean age 332.67 days; *SD:* 45.95 days). A total of 6 infants were excluded from the final sample due to prematurity (<37 weeks of gestation; n = 3) or crying/fussiness (n = 3) during the experimental session. All infants included in the study had normal weight at birth (>2500 gr) and no history of developmental delay. Infants were recruited from the city of Granada (Spain) and surrounding areas by means of adverts at the University of Granada webpage and local newspapers as well as by distributing information sheets among local nurseries, covering various districts of Granada differing in socioeconomic background. Informed written consent was obtained from the infants’ parents or legal guardians.

### Procedure

All infants participating in the study conducted two experimental tasks: attention-switching and emotional disengagement tasks, in which their gaze was monitored with an eye tracker device. The entire experimental session was about 30 minutes long, including baby’s acclimating time with the lab setting, tasks performance, and a brief break between tasks. The experiment was conducted in a semi-dark room. Infants were seated on the caregiver’s lap, in front of the display screen at approximately 60 centimetres from the monitor. Parents were asked to avoid interacting with their infants during the experimental tasks. The experimenter monitored infants’ performance from a contiguous room. The attention-switching task was presented always first, followed by the emotional disengagement task. Parents were provided with a description of the study and were asked to sign the consent form at the beginning of experimental session. Parents received a report with their infant individual results and a 10 € voucher for educative toys in appreciation for their participation in the study. The procedures of the study complied with the guidelines of the Declaration of Helsinki for research with human subjects, and ethical approval for the study was granted by the University of Granada’s ethics committee.

### Apparatus

#### Eye tracker

SensoMotorics Instruments (SMI) corneal-reflection eye tracker RED 250 with iView X Hi-Speed system^44^ was used to record infants’ looking behaviour. The system has a temporal resolution of 250 Hz and a spatial resolution of 0.03°according to manufacturers. Stimuli were displayed in a 1024 × 768 pixel 19-inch monitor (60 Hz). SMI’s Experiment Centre software^44^ was used to control presentation of the stimuli. A 5-point calibration was performed before starting each task. Calibration points were located at the four corners and the centre of the screen. We used colourful looming stimuli with funny sounds to make the calibration procedure child-friendly and grab infants’ attention more easily. Saccades and fixations were computed according to the following parameters: peak velocity threshold = 40°/s; minimum fixation duration = 50 ms.

### Experimental tasks

#### Attention-switching task

The attention-switching task was similar to the task used in a previous research by Kovács and Mehler^27^. Infants saw two white boxes (size: visual angle of 18° × 18°) in a black background presented at either side of the screen at 15° eccentricity. These boxes remained visible throughout the entire trial. An animated star with music was presented in the centre of the screen to attract babies’ attention at the beginning of each trial. The trial started automatically once the baby looked to the attractor for at least 200 milliseconds. After one second delay (anticipatory period), an animated cartoon accompanied by funny sounds appeared in one of the boxes and remained visible for 2 seconds. The initial location of the cartoon (left or right) was counterbalanced across participants. After 9 trials appearing in the same place, the cartoon appeared in the opposite side for another 9 trials. Babies who completed less than 50% of trials in each block or had poor-quality data were excluded from final analyses (n = 8). We computed the proportion of looks to any of the boxes during the anticipatory period. Anticipatory looks that occurred in the first 200 milliseconds after the onset of peripheral target were excluded, as they do not represent a real expectation^45^. Two 21° × 19° areas of interest (AOI) were defined, covering both the left and right box. Only trials with direct looks to one of the two boxes were included in subsequent analyses. Anticipatory looks in the post-switch block to the box in which the animated cartoon appeared during block 1 were considered as perseverations. The percentage of perseverations per participant was calculated as an index of attention flexibility.

#### Emotional disengagement task

The emotional disengagement task was similar to the one developed by Peltola *et al*.^14^. Happy, fearful and neutral faces of two different identities (female and male) from NimStim set^46^ were presented to babies in a computer monitor. Scrambled faces were presented as a control condition. All faces subtended a visual angle of 15.2° × 11.1°. Each trial started automatically after babies looked at a centrally located attention grabbing stimuli for at least 200 milliseconds. Then, a face from any of the 4 experimental conditions appeared randomly on the centre of the screen. A second later, a new stimulus (peripheral target) appeared 13.6° from the central face either on the right or the left side of the screen. The peripheral target consisted of either a black and white check-board pattern or vertically arranged circles (15.4° × 4.3° size). Both stimuli, the face and peripheral target, remained for 2 more seconds. The complete task involved a total of 32 trials. If babies became fussy or bored, the experimenter stopped the task. Only infants that completed at least 4 trials per condition were included in the analyses (n = 55). A trial was considered valid if the infant looked at the central face and remained looking at the screen during the 2 seconds that the peripheral target was present (mean = 6.8, *SD* = 1.37 valid trials, no differences in the number of trials per condition; *F* (3,162) < 1). For analysing gaze, two AOIs were defined: an AOI of visual angle of 17.3° × 6.1° covering either the left or right peripheral target and another one of visual angle of 17.5° × 13.2° covering the central face. As a measure of disengagement, we subtracted the total fixation time to the peripheral target from fixation time to the face for every condition. Larger scores indicate greater difficulty to disengage from faces.

### Questionnaires

#### Temperament questionnaire

After completion of the experimental session, parents were asked to complete the Spanish version of the Infant Behaviour Questionnaire Revised (IBQ-R)^31^. This questionnaire measures temperament in 14 scales grouped in 3 factors: Surgency/Extraversion (SUR), Negative Affectivity (NA) and Orienting/Regulation (REG). Parents were asked to rate the frequency of some infant’s behaviours in different situations during the previous week or 2 weeks in a 7-point scale. Cronbach’s alphas were above 0.70 for all the scales.

#### Socio-economic status measurement

To calculate an index of familial SES we asked parents about their educational level, professional occupation and family income. The educational level was rated from 1 (no formal education) to 7 (postgraduate), professional occupation from 1 (unemployed) to 9 (manager) according to the nine-point scale of the National Classification of Occupations of the National Institute of Statistics of Spain^47^, and income to need ratio (i.e. total annual income divided by national poverty threshold) was calculated as a measure of family income. Scores were z-transformed and averaged in a unique index of SES. Descriptive data of our sample are presented in Table 1.

**Table 1:** Descriptive statistics of all measures included in the study.

			Valid n	Min	Max	Mean	SD
Experimental tasks	% Perseverations in attention-switching task		59	0	100	52.37	32
	Disengagement from face (ms)	Fear	55	252	2631.17	1431.77	618.97
		Happy	55	8.56	2545.41	1213.22	659.31
		Neutral	55	220.71	2449.98	1201.98	541.63
		Control	55	3.97	2240.66	849.40	535.17
SES	SES index (z-scores)		65	−1.32	1.30	0.12	0.67
	Parents Occupation (1–9)		65	1	7.5	5.04	1.23
	Parents Education (1–7)		65	1	7	5.54	1.12
	Family Income to need ratio		65	0.21	3.77	2.04	0.99
Temperament (raw scores)	NA		65	2.96	5.26	3.98	0.48
	SUR		65	4.05	6.58	5.32	0.59
	REG		65	3.62	6.28	5.01	0.56

### Data availability

The datasets generated during and/or analysed during the current study are available from the corresponding author on reasonable request.

## Results

### Attention flexibility: attention-switching task

The proportion of correct anticipations significantly decreased in block 2 (*M* = 47.63, *SD* = 32.23) compared to block 1 (*M* = 77.82, *SD* = 29.16; *t*(58) = 7.35, *p* < 0.001). The percentage of perseverations in block 2 (*M* = 52.37, *SD* = 32.23) was and used as the index of attention flexibility in subsequent analyses.

### Disengagement of attention: Emotional disengagement task

We ran repeated-measures ANOVA to test the effect of emotion of the face in disengagement. We found a significant effect of Emotion (*F*(3,162) = 25.68, *p* < 0.001, *η*_*p*_^2^ = 0.32). Planned comparisons showed that disengagement was easier for non-face stimuli than for fearful (*F*(1,54) = 65.96, *p* < 0.001), happy (*F*(1,54) = 27.74, *p* < 0.001) or neutral faces (*F*(1,54) = 32.77, p < 0.001). There were no differences in disengagement between happy and neutral faces (*F*(1,54) = 0.22; *p* > 0.05), whereas disengagement was more difficult for fearful faces compared to neutral (*F*(1,54) = 18.48, *p* < 0.001), or happy faces (*F*(1,54) = 9.43, *p* < 0.01).

### Age and gender

Pearson’s correlations revealed that age was uncorrelated to perseverations in the shifting task or disengagement from fearful faces (*r* = −0.01, *p* = 0.97 and *r* = 0.05, *p* = 0.69 respectively). Infants’ gender was coded as a dummy variable as follows: 0 = male, 1 = female. Gender was unrelated to performance in the shifting task (*r* = 0.05, *p* = 0.69) but associated with disengagement from fearful faces in such way that female infants had the greater difficulty to disengage from fearful faces (*r* = 0.27, *p* = 0.04).

### Correlations between variables of interest

**Figure 1:**
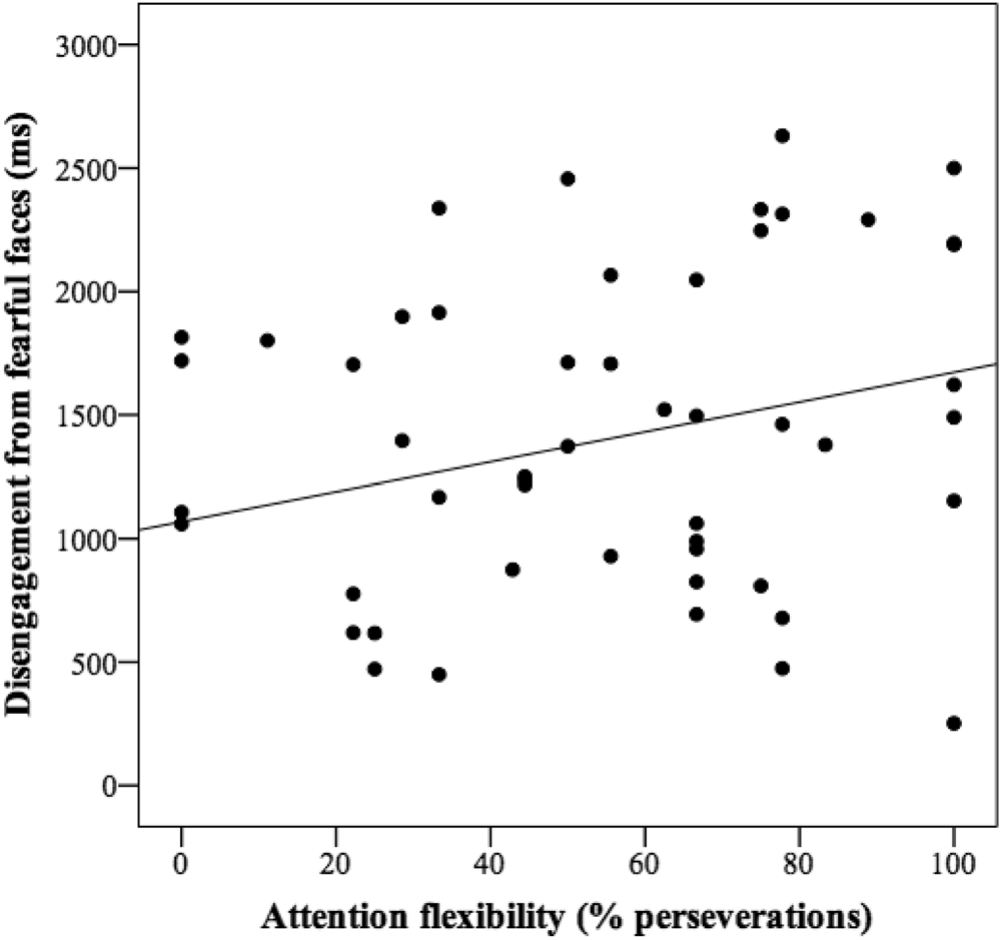
Correlation between disengagement of attention from fearful faces and attention flexibility (% perseverations in attention-switching task).

**Figure 2:**
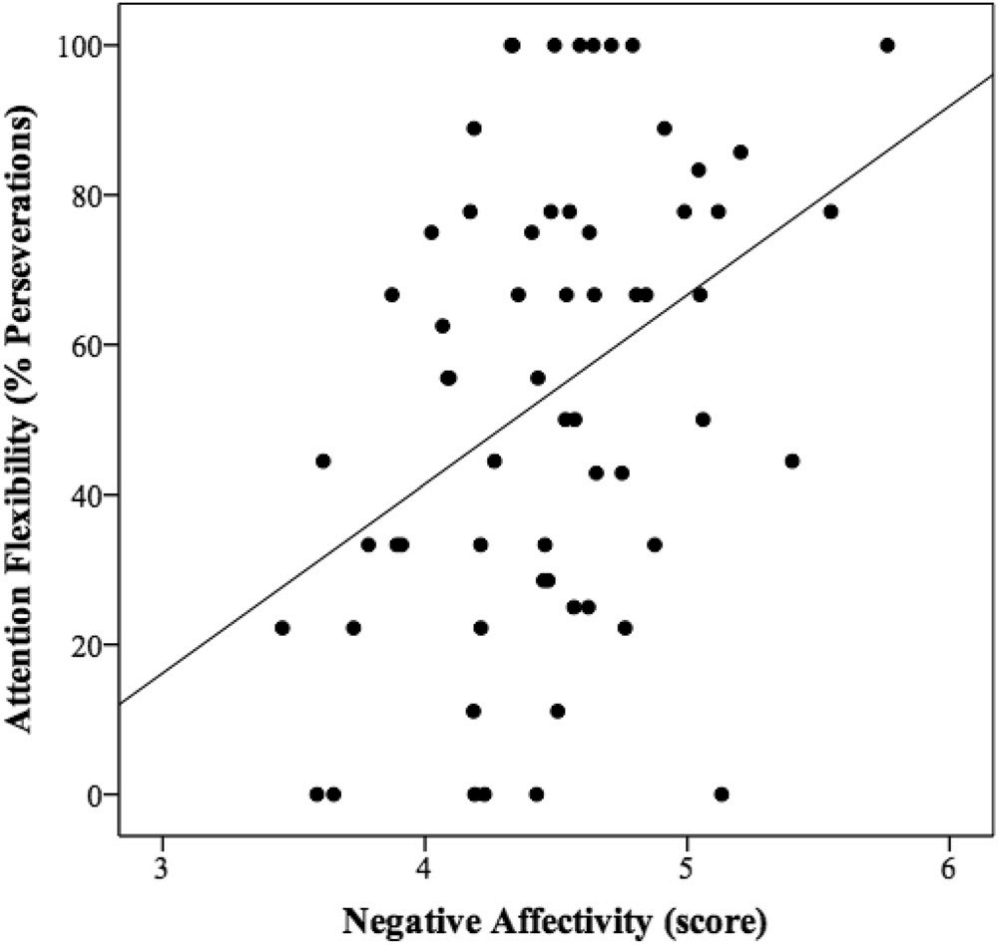
Correlation between attention flexibility (% perseverations in attention-switching task) and Negative Affectivity.

**Figure 3:**
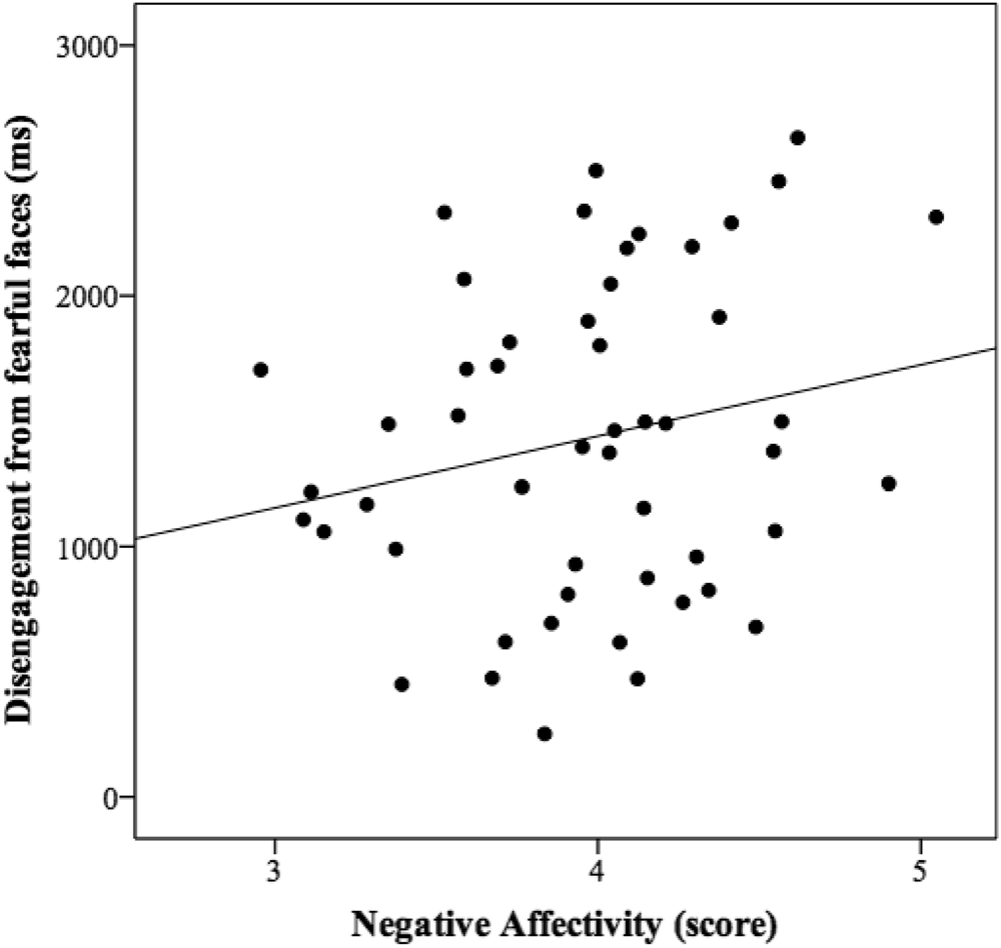
Correlation between disengagement of attention from fearful faces and Negative Affectivity.

**Figure 4:**
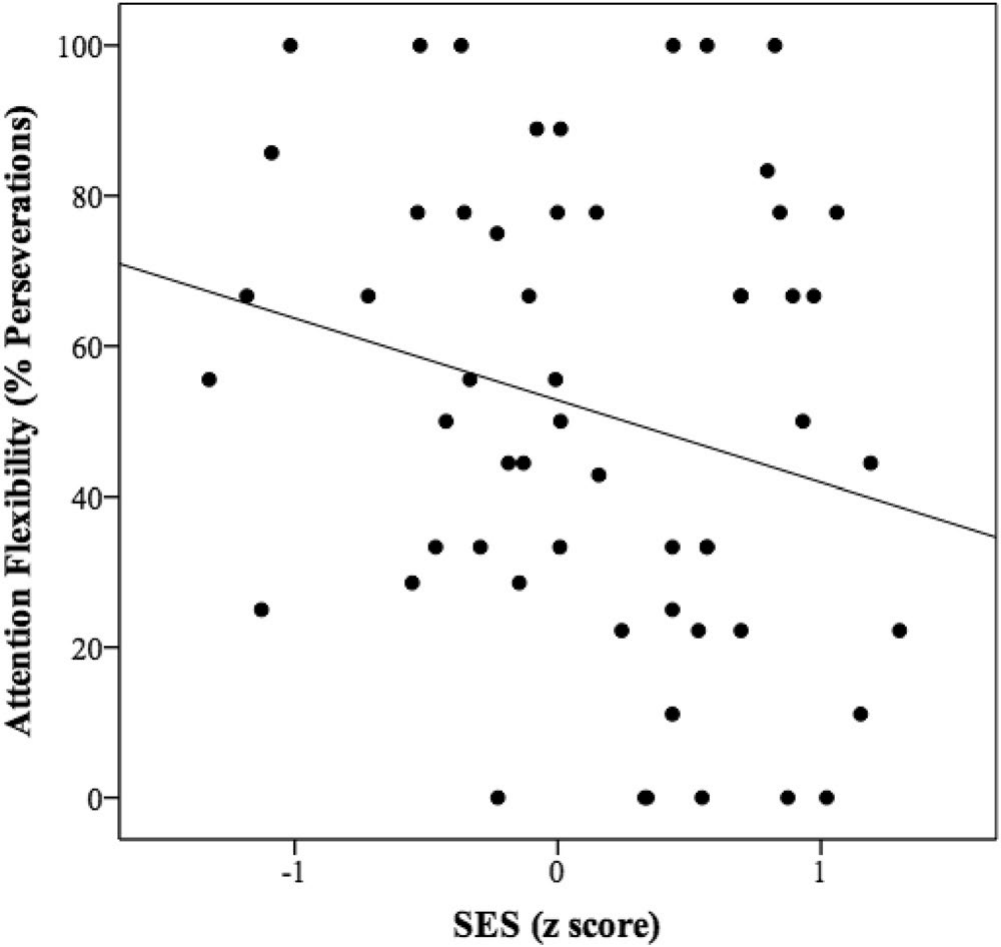
Correlation between attention flexibility (% perseverations in attention-switching task) and SES.

As we were interested in understanding individual differences, partial correlations controlling by age and gender were performed to test inter-correlations among measures of disengagement, attention flexibility, temperament and SES. We found that the percentage of perseverations in the switching task was significantly correlated with difficulty to disengage from fearful faces (*r* = 0.25, *p* < 05; Fig. 1), but did not reach the significance level for happy (*r* = 0.22, *p* = 0.08) and neutral faces (*r* = 0.22, *p* = 0.08), and was also unrelated to the disengagement from control stimuli (*r* = −0.00, *p* > 0.1). In addition, we found a significant negative correlation between SES and NA (*r* = −0.31, *p* = 0.01), however SES was not significantly related to neither SUR (*r* = −0.15, *p* = 0.13) nor REG (*r* = −0.09, *p* < 0.24). Table 2 summarizes correlations of temperament and SES with the performance in the switching and disengagement tasks. With regard to temperament, results revealed that only NA was associated with performance in both tasks. NA was positively related to proportion of perseverations in the switching task (*r* = 0.39, *p* = 0.001; Fig. 2), and difficulty to disengage from fearful faces (*r* = 0.21, *p* < 0.05; Fig. 3). Finally, SES was negatively correlated to perseverations in the switching task (*r* = −0.23, *p* < 0.05; Fig. 4). Although no significant relationship was found between the general index of SES and performance in the emotional disengagement task, family income was negatively related to difficulty of disengagement from fearful (*r* = −0.26; *p* = 0.05), happy (*r* = −0.39, *p* < 0.01), and neutral faces (*r* = −0.30, *p* < 0.05).

**Table 2:** Partial correlations controlling by age and gender among attention regulation, temperament and SES measures.

		Experimental tasks				
		% Perseverations in attention-switching task	Disengagement from faces (ms)			
			fear	happy	neutral	control
SES	SES index	−0.23*	0.05	−0.13	−0.12	−0.01
	Parents Occupation	−0.28*	0.09	−0.07	−0.01	0.01
	Parents Education	0.01	0.08	0.20	0.06	0.13
	Family Income	−0.22^#^	−0.26*	−0.39**	−0.30*	−0.14
Temperament (raw scores)	NA	0.39**	0.27*	0.07	0.07	0.15
	SUR	0.04	−0.07	0.09	0.07	0.16
	REG	−0.15	−0.06	−0.06	−0.08	0.09

**p < 0.01; *p < 0.05; ^#^*p* < 0.10.

### Regression analyses

We ran regression analyses to examine the joint contribution of SES, NA and attention flexibility to disengagement from fearful faces. Analyses were performed with the macro PROCESS for SPSS^48^. Confidence intervals were calculated for 5000 bias corrected bootstrap samples. Age and gender were introduced as a covariate in all tested models.

**Figure 5:**
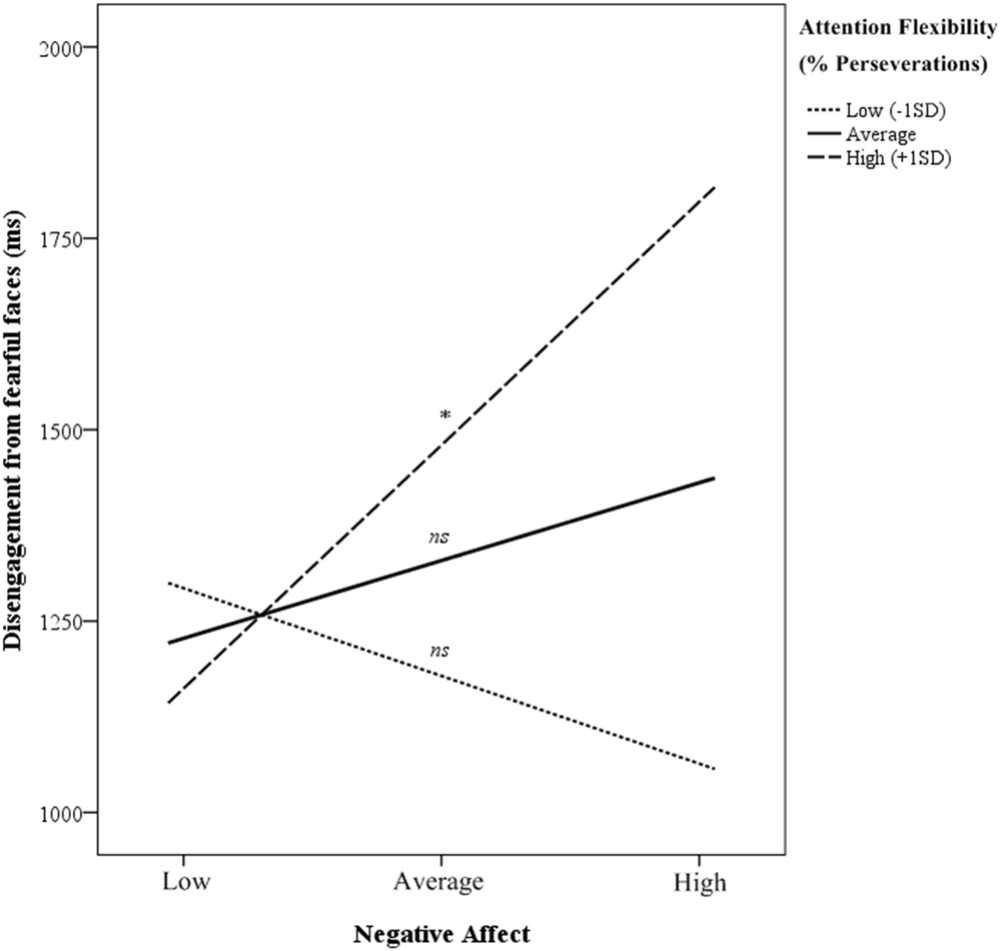
Moderation effect of attention flexibility (percentage of perseverations in the attention-switching task) on the relationship between disengagement from fearful stimuli and NA. Low and high levels of the variables refers to values from 1 SD either below or above the mean. Notice that larger proportions of perseverations indicate poorer attention flexibility. *p < 0.01.

In order to test the hypothesis that attention flexibility moderates the effect of NA to explain difficulty to disengage from fearful faces, we ran moderation analyses following Susa *et al*.^35^ and Lonigan and Vasey^34^. Results are presented in Table 3. The overall model was significant (*F*(5,46) = 2.69, *p* < 0.05, *R*^2^ = 0.23) with the interaction between perseverations in the switching task and NA significantly predicting disengagement from fearful faces (*b* = 14.17, p < 0.05). Adding the interaction term to the model significantly increased the proportion of explained variance (*ΔR*^2^ = 0.09, *F*(5,46) = 5.07, *p* < 0.05). To facilitate interpretation of the moderation effect, the relationship between variables is plotted in Fig. 5. Difficulty to disengage attention does not change as a function of NA for infants with high (low percentage of perseverations; *b* = *−*245.65, *t*(46) = −1.07, *p* = 0.29) or average (*b* = 236.08, *t*(46) = 1.17, *p* = 0.25) attention flexibility, whereas difficulty to disengage from fearful faces significantly increases as a function of NA in the case of infants with poor attention flexibility skills (with high proportion of perseverations in the switching task; *b* = 607.29, *t*(46) = 1.98, *p* < 0.05).

**Table 3:** Moderation analysis testing the moderation of NA in the relationship between Attention Flexibility (% perseveration in attention-switching task) and Disengagement from fearful faces.

DV: Disengagement from fearful faces	*R* ^2^	*B*	*t*	*95% CI*
	0.23*			
Perseverations in attention-switching task (%)		4.98	1.57	[−1.43, 11.40]
NA		201.50	1.03	[−193.16, 596.17]
Perseverations in attention-switching task (%) × NA		14.17	2.25*	[1.49, 26.85]
Age		−19.68	−0.30	[−150.45, 111.10]
Gender		398.67	2.07*	[10.54, 786.47]

Moderation was controlled by age and gender (coded as 0 for male, 1 for female). Significance levels: *p < 0.05.

**Figure 6:**
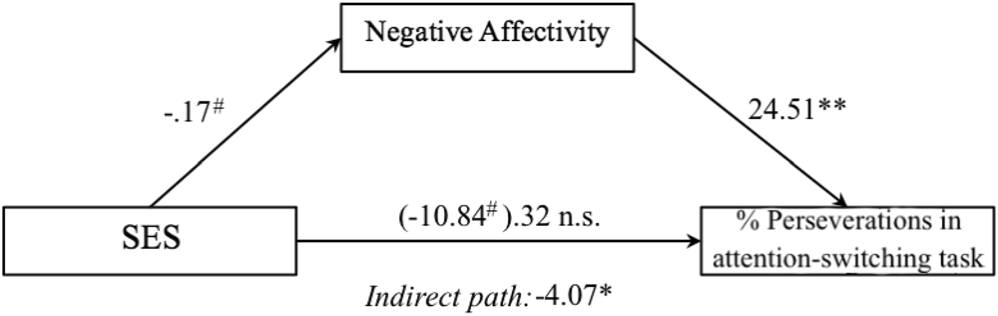
Role of NA as a mediator of SES effect on attention flexibility as measured by the percentage of perseverations in the attention-switching task. Values represent unstandardized regression coefficients. Value inside parentheses indicate coefficient for the direct path from SES to attention flexibility before controlling for NA. *p < 0.05, **p < 0.01, ^#^*p* < 0.10.

To examine the contribution of SES and temperament to individual differences in attention flexibility and disengagement of attention, we firstly conducted a mediation analysis in order to test whether NA was mediating the association of SES and attention flexibility. This analysis revealed that SES marginally predicted NA (*b* = −0.17, *t*(55) = −1.83, *p* = 0.07) and percentage of perseverations in the switching task (*b* = −10.84, *t*(55) = −1.71, *p* = 0.09). Introducing together NA and SES in the model significantly predicted percentage of perseverations *F*(3,55) = 2.77, *p* < 0.05, *R*^2^ = 0.18). However, SES did not longer predict perseverations in the switching task after controlling for NA (*b* = −6.77, *t*(55) = −1.09, *p* > 0.05), whereas NA remained significant (*b* = 24.51, *t*(55) = 2.65, *p* < 0.01). This result, illustrated in Fig. 6, indicates that SES shows an indirect contribution to attention flexibility (i.e. rate of perseverations in the switching task), being mediated by NA. We estimated the coefficient for this indirect effect at a confidence interval level of 95% (p < 0.05), resulting in a value of *b* = −4.07; *CI* [−11.45, −0.07]. Data did not support a moderation model, as the SES x NA interaction did not predict attention flexibility (*b* = 6.43, *p* = 0.62). On the other hand, neither mediation nor moderation models significantly predicted disengagement from fearful faces (*b* = −19.01, *ns* and *b* = 113.49, *ns* for the indirect effect in mediation and interaction term in moderation models respectively).

## Discussion

The main purpose of the current research was to study individual differences in regulation of attention in infancy by examining infants’ ability to disengage attention from threat-related stimuli (fearful faces), attention flexibility and the influence of temperament and SES. Results indicate that disengagement from threat-related stimuli is related to infants’ NA and attention flexibility skills. We found a positive relationship between difficulty to disengage and temperamental NA. Further, the relationship between disengagement from threat-related stimuli and NA was moderated by attention flexibility indicating that infants with poorer attention flexibility showed the greatest difficulty to disengage. In addition, SES did not affect disengagement from threat-related stimuli, although low-SES was associated to poorer attention flexibility.

Consistent with previous results, we found that infants showed greater difficulty to disengage from fearful than happy or neutral faces^14–19^. Our data further indicate that infants with higher NA tend to show greater difficulty to disengage from threat-related stimuli. This is in agreement with evidence from another infant study that used the same experimental paradigm^16^. Inconsistently to these results, one recent study failed to demonstrate the relationship between NA and infants’ disengagement from angry faces, also considered as threat-relevant^49^. Although some studies have found a general attention-bias to initial orienting to negative valence stimuli with no differences between angry and fearful faces^50^, other studies suggest that fearful and angry faces are processed in a different way. Both fearful and angry faces convey threat-related information, but differ about the type of information provided. Fearful faces signal the presence of an undetermined threat, whereas angry faces denote a direct intrinsic threat that elicits a withdrawal response^51^. This may explain the disparity of results and point to the need to be more cautious when comparing studies that use fearful and angry faces.

Our findings also confirmed that infants’ attention flexibility moderates the relationship between NA and disengagement from fearful faces. As can be seen in Fig. 5, infants with higher NA levels who also show poor attention flexibility (i.e. high percentage of perseverations) present the greatest difficulty to disengage from fearful faces. However, babies showing good attention flexibility skills (i.e. low or average percentage of perseverations) are better able to disengage from fearful faces independently of NA. These results are in line with previous findings concerning attention bias to threat with older children^34,35^, giving further support to recent models about the development of attention regulation to emotional stimuli in infancy^24^. Similarly, attention flexibility has been demonstrated to play a fundamental role facilitating disengagement from threatening stimuli in individuals with high anxiety, moderating the effect of anxiety in the same way as we observed in infants with high NA^21,52^. Overall, the interaction between temperament and attention flexibility during infancy may constitute a valuable model for predicting future regulation of emotion, determining risk patterns for the development of externalizing and internalizing problems (such us anxiety disorders), and informing initiatives aimed at preventing social maladjustment and psychopathology from very early in development.

Considering the influence of SES, we found that low SES was associated with poorer attention flexibility (i.e. increased perseverations). Lower family income, but not the general index of SES, was also related to poorer ability to disengage from faces, regardless the emotion expression of the face. These results suggest a general effect of family resources over the development of attention flexibility skills. This is consistent with existing literature showing a significant contribution of family SES to the development of executive attention from early^53^ to older ages^54^. Our results are also in agreement with prior studies showing an impact of SES in the performance of infants in the A-not B task, a task targeting babies’ attention flexibility skills^38,39^. However, the role of temperament was not examined in such studies. Our data show that NA is related to poorer attention flexibility. Surprisingly, attention flexibility was unrelated to the regulation/orienting temperamental factor. However, it has been suggested that regulation/orienting as measured with the IBQ-R may capture exogenous mechanisms of attention regulation to a greater extent than infants’ endogenous control of attention^29^. Further analyses including both SES and NA revealed that SES had an indirect effect on attention flexibility mediated by infants’ NA. Consequently, our results do not support a differential susceptibility model^42^. Instead, a mediation model appears more adequate to explain our results. There is evidence that children raised in low SES contexts are more likely to be exposed to adversity, and subsequently to higher stress levels^55^. Increased stress experimented by low-SES children might have an impact on the development of neuroendocrine and autonomic responses to stimulation. In fact, low-SES children show slower autonomic recovery and higher cortisol levels^56,57^, even from infancy^58^, which likely predisposes them to greater temperamental reactivity^59^. Increased levels of NA associated with low SES appear to affect the development of attention flexibility in infants, as suggested by our results. Infants with higher NA may be more likely to inhibit their behaviour in reaction to novel stimuli, which could limit infants’ exploratory behaviour, leading to a lack of flexibility^60^. However, further research is needed in order to test this tentative explanation.

Despite its exploratory nature, this study offers some insight into the developing mechanisms of attention regulation in infancy. Findings from this study evidenced the key role of attention flexibility in the disengagement of attention from threat-related stimuli as early as from infancy. This may translate in the early detection and prevention of risk for anxiety disorders later in development. It has been proposed that enhancing attention control not only improves children’s ability to disengage from negative stimuli, but may also serve as a mean to reduce anxiety symptomatology^61^. Although our findings support this idea, inferences about the directionality of the results were limited as all measures were obtained concurrently in this study. Longitudinal research may serve to shed light about this question. Besides, we regarded disengagement from threat-related stimuli as a rudimentary form of emotional regulation. However, we did not measure the effectiveness of disengagement in reducing distress levels. Including complementary measures addressing infants’ emotional response are needed in order to establish the relationship between regulation of attention to emotional stimuli and the emergence of self-regulation. Additionally, we found a relationship between gender and the ability to disengage from fearful faces. Our data indicate that female infants engage longer with fearful faces than male. This can be interpreted as the result of an enhanced recognition of emotion signals for women compared to men, which is consistent with the literature about gender differences in emotion processing^62^. Our data show that this gender effect is already observable by the end of the first year of life. Previous studies with infants did not explore gender differences in emotion disengagement^14–19^, hence more systematic research on this topic is needed.

Finally, data from this study highlight the importance of considering together the effect of environment and temperament to explain individual differences in the emergence of attention regulation in infancy. An important question to this matter relates to what aspects of the environment are more responsible for shaping infants’ cognitive development. Whereas some authors argue that aggregate measures better represent SES, others call to consider the specific contribution of each component of the SES depending on the outcome measure^63^. In our study, a composite measure of SES was used. However, when looking at the different indicators of SES, family income (for both attention flexibility and disengagement) and occupation (for attention flexibility), but not parental education, show a significant association with infants’ attention performance. This indicates that financial resources of the family may be more critical than other aspects of parenting associated with education in the early stages of babies cognitive development. Economic resources may impact more directly aspects of the home environment such as nutrition, quality of household and neighbourhood environment, caregivers’ stress levels, household relationships, and availability of quality time to spend with the baby. On the other hand, caregivers’ education may be more related to parenting styles and the use of cognitive stimulation strategies. Future research should include more specific information of the characteristics of home environment in order to disentangle the potential differential effects of the various aspects integrated in family SES measure. Likewise, more research is needed in order to identify other variables that are likely contributing to the early development of attention regulation, such as parenting^64^ or genes codifying attention-related neurotransmitters^65^. Studies like the present one contribute in several ways to our understanding of individual differences in attention regulation in early development. Prospective studies addressing regulation of attention from a longitudinal perspective may also serve to trace trajectories leading to different developmental outcomes. This knowledge would enhance prevention by both benefiting the early detection of infants at risk for disorders involving deficits of attention regulation (e.g. anxiety, attention deficit disorders, autism, etc.) as well as enabling the adjustment of interventions to the individual characteristic of children’s and their families.
